# Flexing the Spectrum: Advancements and Prospects of Flexible Electrochromic Materials

**DOI:** 10.3390/polym15132924

**Published:** 2023-07-01

**Authors:** Gulzat Nuroldayeva, Mannix P. Balanay

**Affiliations:** 1 Department of Chemistry, Nazarbayev University, 53 Kabanbay Batyr Ave., Astana 010000, Kazakhstan; 2 Institute of Batteries LLC, 53 Kabanbay Batyr Ave., Astana 010000, Kazakhstan

**Keywords:** flexible electrochromic materials, organic-based electrochromic materials, inorganic-based electrochromic materials, hybrid-based electrochromic materials

## Abstract

The application potential of flexible electrochromic materials for wearable devices, smart textiles, flexible displays, electronic paper, and implantable biomedical devices is enormous. These materials offer the advantages of conformability and mechanical robustness, making them highly desirable for these applications. In this review, we comprehensively examine the field of flexible electrochromic materials, covering topics such as synthesis methods, structure design, electrochromic mechanisms, and current applications. We also address the challenges associated with achieving flexibility in electrochromic materials and discuss strategies to overcome them. By shedding light on these challenges and proposing solutions, we aim to advance the development of flexible electrochromic materials. We also highlight recent advances in the field and present promising directions for future research. We intend to stimulate further innovation and development in this rapidly evolving field and encourage researchers to explore new opportunities and applications for flexible electrochromic materials. Through this review, readers can gain a comprehensive understanding of the synthesis, design, mechanisms, and applications of flexible electrochromic materials. It serves as a valuable resource for researchers and industry professionals looking to harness the potential of these materials for various technological applications.

## 1. Introduction

### 1.1. Understanding Electrochromic Materials: Definition and Working Principles

Electrochromism is a remarkable property of certain materials that sets them apart from other substances [1]. These fascinating electrochromic materials have the unique ability to reversibly change their color or optical properties when exposed to an external electrical stimulus. Through reversible redox reactions within the material, the dynamic nature of electrochromism (EC) enables effective modulation of its light absorption and transmission properties across the electromagnetic spectrum [2]. When an electrical voltage is applied to these materials, which contain certain electroactive components, oxidation or reduction reactions are initiated. As a result, the materials significantly change their physical appearance and alternate between different color states. By precisely controlling these changes and the timing of the changes, the desired effects can be achieved [3]. These unique adaptations have found numerous applications in areas such as the design of advanced structures [4], displays [5], and optical devices [6]. The exceptional properties of these materials are particularly valuable for the development of energy-efficient heat and light transfer systems [7]. They can switch between transparent and colored states, which offers advantages in architecture, automotive, and other industries.

**Figure 1:**
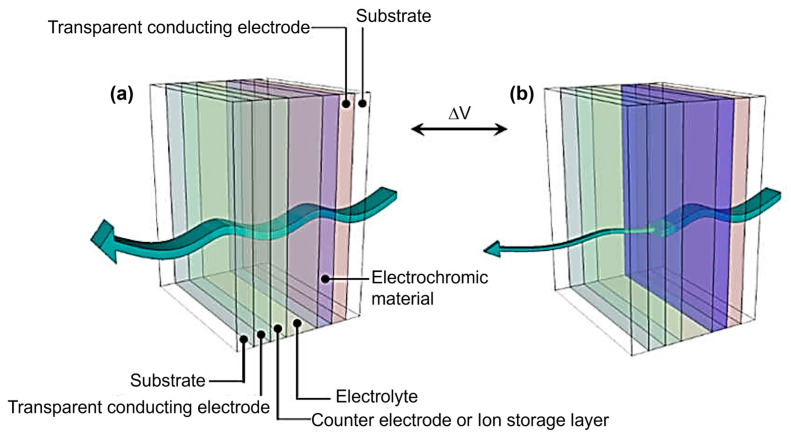
A general schematic diagram of an electrochromic device showing the (**a**) bleached and (**b**) colored states. Adapted with permission from reference [11]. Copyright: the authors, some rights reserved, exclusive licensee MDPI. Distributed under a Creative Commons Attribution License 4.0 (CC BY).

An electrochromic system usually consists of several major components (Figure 1). The electrochromic layer, located at the heart of the system, consists of a material that can undergo a reversible redox reaction when electric current flows through it, resulting in a change in its color state. Adjacent to the electrochromic layer is the ion storage layer or counter electrode, which serves as a reservoir for the ions involved in the redox process and facilitates their movement to and from the electrochromic layer. On either side of these layers are two transparent conductive electrodes responsible for applying the electrical potential to the system. These electrodes allow the ions to move and trigger the color change. It is worth noting that the substrate on which the electrochromic system is built can be either rigid or flexible, depending on the application. Examples of commonly used substrate materials include glass, plastic films, and flexible polymers. Finally, the electrolyte plays a crucial role by providing a medium for ion transport and ensuring the overall electrochemical stability of the system [8,9,10,11].

**Figure 2:**
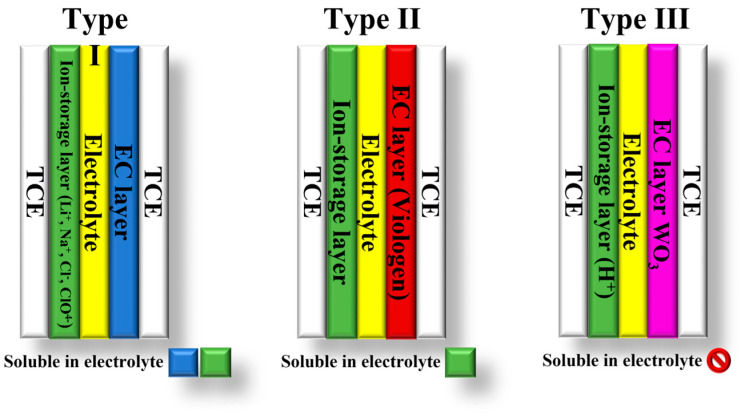
Types of electrochromic devices based on the solubility of the EC components.

The classification of electrochromic devices (ECDs) is based on the solubility of the bleached and colored electrochromic components in the electrolyte layer. There are three types of ECDs: type I, II, and III [1,12,13] (Figure 2). Type I devices are characterized by the fact that both components are soluble in the electrolyte during operation. In contrast, insoluble solids of both components form on the electrodes in type III devices [12]. In these devices, the electrolyte layer does not contain EC materials. Their purpose is to provide counterions for the EC layers to maintain charge neutrality during electrochemical coloration and bleaching. Type II ECDs fall between type I and type III. In type II devices, only the bleached component is dissolved in the electrolyte, while the colored component forms a coating on one of the electrodes [12,13].

By carefully optimizing materials, device configurations, and electrical stimuli, electrochromic materials offer a wide range of applications [14]. One notable example is smart windows [11,15], which can regulate the amount of light entering an outdoor space to improve energy efficiency and occupant comfort. Zhai et al. [16] investigated dual-band electrochromics, i.e., the independent modulation of visible (Vis) and near-infrared (NIR) light transmittance by EC materials. Dual-band electrochromic devices have unique characteristics, such as bistability, low power consumption, and separate control over the visible and NIR regions. Therefore, the development of dual-band EC devices is of great importance in the pursuit of an energy-saving society.

Previous studies have shown the working mechanism of electrochromic materials in supercapacitors, electric double-layer capacitors (EDLCs), and lithium-ion batteries (LIBs) [17,18,19,20,21]. The different electrochemical processes that determine the charge storage properties of batteries and supercapacitors can be distinguished through electrochemical measurements [18]. Unlike batteries, EDLCs respond quickly to voltage changes and have high energy density because no redox reactions occur [19,20]. However, EDLCs have lower energy density because the charges are mainly located on the material surfaces. The main characteristics of EDLCs include a rectangular cyclic voltammogram curve (CV) and a linear discharge curve versus time [21].

Recent research shows that the integration of electrochromic technology with other cutting-edge technologies has significantly accelerated its progress [22,23,24,25]. This integration has expanded the applications of electrochromic technology in various fields. Researchers have explored a novel self-structuring electrochromic device that uses a single layer of multicolor polyaniline (PANI) [26]. Unlike conventional symmetric ECDs with five functional layers, this design features a three-layer structure that includes a PANI EC layer, a quasi-solid-state gel electrolyte, and a conductive layer [27]. The self-structuring ECD exhibits a distinct multicolor and gradient pattern and achieves significant transmission contrast of up to 60% when a horizontal current is applied [26]. To understand this self-structuring behavior, a novel electrochromic mechanism was proposed to highlight the planar motion and redistribution of anions in the PANI layer, which is triggered by a current-driven magnetic effect.

To expand the practical applications of electrochromic devices, the introduction of flexible architectures enables new functions [28]. The development of flexible electrochromic devices (FECDs) has led to electronic skins [29], actuators [30], and wearable electronics [31], expanding the range of their potential applications [28]. FECDs are characterized by their compact size, improved mechanical durability, and multifunctionality, and have attracted considerable attention in both academic and industrial fields [32]. With the increasing demand for portable, flexible, and deformable electronic devices, FECDs play a crucial role in meeting these requirements. Their light weight makes them promising candidates for human–machine interfaces that seamlessly integrate with soft biological tissues, such as the surface of the human body [33].

### 1.2. Background and Significance of Flexible Electrochromic Materials

Flexible electrochromic materials have emerged as a promising class of materials with immense potential for applications in flexible and wearable electronic devices [34]. These materials have the unique ability to change their color or optical properties in response to an external electrical stimulus while maintaining their flexibility and mechanical integrity [35]. The combination of electrochromics and flexibility opens exciting possibilities for the development of advanced technologies that can seamlessly conform to curved surfaces, assume irregular shapes, and cannot be bent or stretched. The field of flexible electrochromic materials has gained significant momentum due to the growing demand for flexible electronics and wearable devices [36]. Conventional electrochromic materials, while efficient at modulating optical properties, often do not provide the flexibility required for these new applications. Therefore, the development of flexible electrochromic materials has become an important area of research to overcome this limitation and open up new opportunities. To achieve flexibility in electrochromic materials, several strategies have been explored. These include the use of flexible substrates, the use of stretchable conductive materials [37,38,39], and the modification of the molecular structure of the electrochromic materials themselves [40,41,42]. These approaches aim to preserve the electrochromic functions while improving the mechanical properties so they can withstand repeated deformations.

The integration of flexible electrochromic materials into wearable devices and smart textiles holds tremendous potential [43,44,45]. Imagine garments that can change their color or pattern or adjust their transparency depending on environmental conditions with a simple touch. Such applications have the potential to revolutionize the fashion industry and enable interactive and customizable designs. In addition, flexible electrochromic materials could prove invaluable for flexible displays [41], electronic paper [46,47], and implantable biomedical devices [48,49], where adaptability and mechanical robustness are critical requirements. This review addresses the exciting field of flexible electrochromic materials and examines their synthetic methods, structural design, electrochromic mechanisms, and current applications. We also discuss the challenges associated with achieving flexibility in electrochromic materials and present strategies to overcome them. In addition, we highlight recent advances and promising directions for future research to encourage further innovation and development in this rapidly evolving field.

### 1.3. The General Structure of Flexible Electrochromic Materials

**Figure 3:**
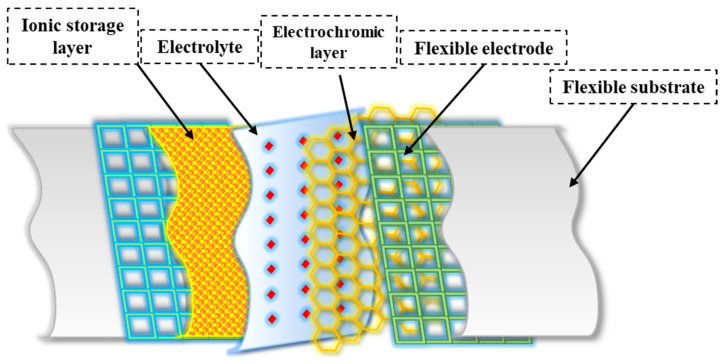
General structure of flexible electrochromic materials.

Multiple layers play an essential role in the overall structure of flexible electrochromic materials and enable the desired electrochromic behavior. While the specific structure varies depending on the material system and device structure (see Figure 3), here is a general framework for flexible electrochromic materials:

- The substrate serves as a flexible base to which other layers are applied. It provides mechanical support and flexibility to the electrochromic device. Compared to rigid substrates, flexible substrates offer several advantages, including increased flexibility, portability, and lower cost [25,50]. These properties allow flexible electrochromic devices to maintain their electrochromic properties even when bent, twisted, or stretched [25]. For transparent FECDs, an ideal flexible transparent substrate should have high optical transparency, excellent resistance to environmental and chemical factors, and mechanical flexibility. Reflective FECDs, on the other hand, use flexible substrates made of materials such as nylon/Au or PET /Au, which serve as reflectors [51,52]. In addition, polymers (e.g., polyethylene terephthalate or PEN [53], polyimide [54], and flexible glasses (e.g., thin soda-lime glass [55]) can also be used as flexible substrates.
- The transparent conductive electrode is usually located on the substrate and serves as a current collector for the electrochromic layer. It allows the electric current to pass through and remains transparent in the visual spectrum. Several critical factors affect the performance of FECDs with transparent conductive electrodes (TCEs). These include low resistivity, high transparency, a wide potential window, improved chemical and electrochemical stability, and increased resistance to deformations such as bending, stretching, and folding [56]. Indium tin oxide (ITO) [57] and fluorine-doped tin oxide (FTO) [58] are widely used for the fabrication of electrochromic devices due to their low resistance and high transparency. However, the adhesion of ITO to flexible substrates often leads to significant degradation of the dyeing efficiency and optical contrast of FECDs after several bending cycles [59]. In addition, the brittleness and high cost of ITO pose challenges to its suitability for flexible applications and commercialization of FECDs [41]. Therefore, alternative flexible electrode materials such as conducting polymers [60], carbon nanotubes [61], graphene [62], metal nanowires [63], and grids [64] have been thoroughly investigated as potential replacements for the traditional ITO /FTO to improve the overall flexibility of the devices.
- The electrochromic layer is the active layer responsible for reversible color change or a change in optical properties. It undergoes redox reactions in response to an applied electrical potential. The electrochromic layer, which consists of an electrochromic material, plays an important role in flexible electrochromic devices [65]. This layer enables the reversible electrochemical redox process that allows visual manipulation. The EC films require properties such as strong ionic and electron conductivity, a significant optical difference between the dyeing and bleaching states, high dyeing efficiency, and consistent cycling stability [66]. Flexible EC films are particularly valuable for FECDs, and organic EC materials, especially conjugated polymers, have found wide application [67]. In addition, hard inorganic materials can be used to fabricate EC films whose flexibility is enhanced by selective morphology and structural design [68]. The choice of electrochromic material depends on criteria such as the desired color range, response time, durability, and device compatibility [69]. In addition to the primary electrochromic layer, the ion storage layer plays a crucial role in maintaining the stability of FECDs. This layer cooperates with the primary electrochromic layer to facilitate the reversible exchange of small ions and charge-balancing electrons between the electrodes and the electrolyte layer [9]. It effectively reduces the accumulation of small ions on the electrode surfaces and prevents their injection into the electrodes, which is critical for device performance and lifetime. Complementary electrochromic materials are often used as an alternative to the ion storage layer to improve the optical modulation and coloring efficiency of electrochromic devices [2]. This substitution allows the ion storage layer to change color when the devices are dyed, which improves the overall performance of the devices.

### 1.4. Overview of the Application and Potential Benefits of Flexible Electrochromic Materials

Electrochromic devices include a range of technologies that use the electrochromic effect to modulate color or optical properties in response to an electric field [70]. These devices have attracted considerable attention due to their applications in various fields, including smart windows, displays, and optical filters. Understanding the different types of electrochromic devices provides valuable insight into their unique functions and potential applications.

- An important application of electrochromic devices is the development of smart windows [71]. These windows contain thin films or coatings of electrochromic materials that can switch between transparent and opaque states. By applying a voltage, these windows can dynamically control the amount of light and heat entering a building or vehicle, providing energy-efficient solutions for lighting and air conditioning.
- Electrochromic displays are another fascinating application of electrochromism [72]. These displays use electrochromic materials that can change color or opacity to produce visual information. Electrical signals selectively drive individual pixels or segments to produce the desired image or text information. The advantages of electrochromic displays are their low power consumption and high contrast capability, making them suitable for e-readers and low-power electronic signage.
- Electrochromic mirrors are used in automotive applications to reduce glare from the headlights of following vehicles [73]. These mirrors consist of an electrochromic layer sandwiched between two transparent conductive layers. When an electrical voltage is applied, the mirror darkens, reducing the intensity of reflected light and improving driver visibility and safety.
- Electrochromic materials can be used to fabricate EC sensors that detect and quantify various analytes [74]. In these sensors, a reaction usually occurs between the analyte and a particular electrochromic material, resulting in a color change that can be measured and correlated with the concentration of the analyte. Electrochromic sensors are used in environmental monitoring, food quality control, and medical diagnostics.
- Recent advances have enabled the integration of electrochromic materials into textiles, resulting in smart fabrics with color-changing capabilities [75]. These fabrics can be used for wearable technology, fashion, or artistic installations to achieve dynamic and interactive designs. The color and appearance of the fabric can be changed by applying an electrical potential, allowing for a customized and customizable esthetic experience.

Various flexible electrode materials and design methods have enabled the fabrication of flexible and stretchable electrochromic devices with excellent mechanical and photoelectric properties. Indium tin oxide on polyethylene terephthalate (ITO/PET) is widely used as a flexible conductive material. In addition, new transparent conductive electrodes such as carbon nanotubes, graphene, and metal-based nanomaterials such as Ag/Au nanowires and soft conductive polymers have emerged as viable alternatives for fabricating flexible electronic devices. Through the use of various electrochromic materials and physical or chemical processes, flexible electrochromic electrodes with electrochromic functionality have emerged, building on advances in flexible electrodes. These advances have paved the way for the development of flexible electrochromic devices.

## 2. Types of Flexible Electrochromic Materials

The development of flexible electrochromic devices is critical to meet the need for multifunctional devices for various applications. Electrochromic materials can be divided into three types: inorganic, organic, and hybrid systems [76]. Inorganic materials are known for their stability and reliability. However, they often suffer from limitations such as limited color options, slow response times, and high production costs [77]. On the other hand, organic electrochromic materials offer advantages in terms of processability, a wide range of colors, and fast response times [78]. However, they may also have disadvantages in terms of temperature resistance, chemical stability, and device durability [79]. Hybrid materials combine the best properties of different material types, resulting in improved electrochromic performance [16]. By combining different materials, hybrid systems outperform single-component materials by exhibiting greater color contrast, faster switching rates, and improved stability [36,80].

### 2.1. Inorganic-Based Flexible Electrochromic Materials

Inorganic electrochromic materials consist mainly of transition-metal oxides. These materials have the properties of wide-bandgap semiconductors and can be completely transparent in the visible region [15]. Their electron shells are inherently non-stable, so metal ions with different valence states can coexist. When exposed to external electric fields, the movement of ions and charge-balancing electrons within these transition-metal oxides leads to changes in the valence electron configuration of the metal ions. This change directly affects the absorption properties of the material and results in visible color changes. Inorganic electrochromic materials can be divided into two categories based on their coloring technique: cathodic electrochromic materials and anodic electrochromic materials. Cathodic electrochromic materials [81], including WO_3_ [82] and MoO_3_ [83], produce a vivid color state due to the injection of ions and charge-balancing electrons. Conversely, anodic electrochromic materials such as NiO_x_ and Ni(OH)_2_ exhibit a colored state by extracting ions and charge-balancing electrons under the influence of an applied electric field [84,85,86].

#### 2.1.1. Examples and Case Studies of Inorganic-Based Flexible Electrochromic Materials

**Figure 4:**
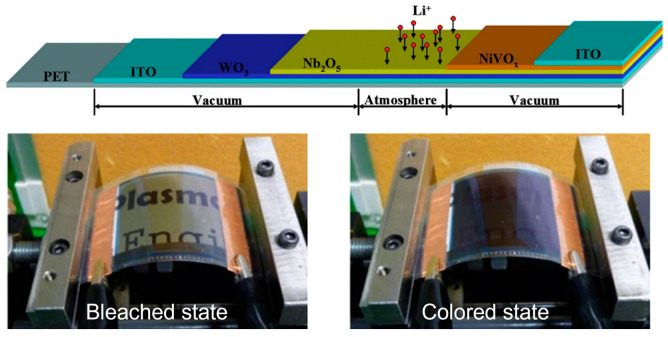
(**Top**) Layered monolithic structure of flexible electrochromic device. (**Bottom**) Experimental setup for conducting bending tests on ECDs in both bleached and colored states. Adapted with permission from reference [80]. Copyright (2016), Elsevier.

One of the most commonly used types of inorganic electrochromic materials is WO_3_, which is very promising due to its exceptional electrochromic, photochromic, and gasochromic properties [87]. For example, researchers have integrated a monolithic solid-state ECD with an effective area of 24 cm × 18 cm on a PET substrate via magnetron sputter coating [80]. The device consists of an ITO/WO_3_/Nb_2_O_5_/NiVO_x_/ITO configuration (Figure 4). The electrochromic properties and flexural strength of this material have been thoroughly investigated. According to these studies, the ECD exhibits fast response times, with a staining response time of six seconds at an applied voltage of −3 V and a bleaching response time of five seconds at an applied voltage of +3 V. The optical transmittances of the device in the bleached and dyed states, measured at a wavelength of 633 nm, were determined to be 53% and 11%, respectively.

Some authors have proposed preparing thin WO_3_ films using the sol–gel method and dip-coating technique on a flexible ITO/PET substrate at different deposition rates [88]. The results showed that the deposition rates affected the solid-state diffusion of Li^+^ ions into the WO_3_ matrix, which worked well during the reduction process. At higher applied potentials, potentiostatic intermittent titration measurements showed an increase in the diffusion of Li^+^ ions. In addition, the film showed exceptional cyclic stability over 200 voltametric cycles.

The method presented by Kim et al. [89] has the advantage of using a low-temperature process that allows flexible electrodes to be incorporated into polymer substrates. Taking advantage of this property, they have effectively demonstrated the implementation of FECDs on plastic. This result greatly expands the application range of WO_3_-based ECDs for flexible electronics. It should be noted that the manipulation of micromorphologies in electrochromic materials can improve their optical modulation capability and mechanical flexibility when incorporated into FECDs [90]. Moreover, these materials have remarkable properties, such as a high specific surface area, permeable channels, and favorable compatibility with the substrate [91].

Another promising material is MoO_3_, which has attracted much interest due to its remarkable photochromic and electrochromic capabilities [83]. The continuous framework and layered structure of MoO_3_ enable efficient ionic movement inside, resulting in remarkable electrochromic performance characterized by fast response times, low starting potential, high staining efficiency, and fine color shifts [92]. Several researchers have successfully developed high-performance electrochromic devices using dispersible mRGO-MoO3-x nanohybrids [93]. These nanohybrids are fabricated by integrating nanoparticles on graphene sheets through a solvothermal reaction, which enables their effective use in electrochromic applications. A noteworthy aspect is that the mRGO-MoO3-x nanohybrids can be easily deposited on substrates using a device-free Langmuir–Blodgett film deposition method, resulting in remarkably thin films. The combination of the enhanced interaction between mRGO-MoO_3-x_ and the ultrathin film structure contributes to the excellent electrochromic properties of mRGO-MoO_3-x_ Langmuir–Blodgett films, including high dyeing efficiency and impressive reversibility even after repeated cycles. These films show great potential for smart window applications.

Most previous research has used a novel and versatile ITO-free MoO_3_/Ag/MoO_3_ (MAM) structure as a highly stable and flexible material for transparent electrodes and electrochromic applications [94]. The researchers used a dielectric–metallic–dielectric (DMD) [59] structure to fabricate a transparent electrode that exhibits high optical transmittance and uniform conductivity. In contrast to the conventional method of modifying MoO_3_ at ITO, the use of MAM films brought several advantages, including faster response times, greater optical contrast, higher staining efficiency, and improved electrochemical and mechanical durability.

Nickel oxide (NiO_x_) is a well-known inorganic material for electrochromic applications, being known for its ability to modulate optical transmittance through electrochemical oxidation and reduction [85]. In addition, NiO_x_ is inexpensive and readily available, which makes it a promising candidate for future large-scale fabrication of EC materials [86]. The excellent electrochromic performance and mechanical stability of NiO_x_ also enhance its potential for use in flexible electrochromic devices. More specific research issues were explored in a study by Kim et al., who employed a low-cost sol–gel spin-coating method for NiO powder to develop NiO-based ITO and flexible devices [82]. The NiO powder is prepared using a simple solid-state process. By incorporating a WO_3_ cathodic electrode at low voltage, the flexible NiO device shows improved electrochromic performance and higher stability. These results show that NiO/WO_3_ can be used as a flexible device for applications such as smart windows and optical devices.

Finally, another promising research direction has demonstrated a novel nanostructured film, nickel carbonate hydroxide (NCH), consisting of a compact seed layer under a porous nanowire layer [95]. This unique NCH film exhibited an exceptional capacitance of over 170 mF/cm^2^ (at a scan rate of 10 mV/s), exceeding the capacitance of the best WO_x_-based energy storage device by 66%. In addition, the NCH film exhibited a high optical contrast of about 85% at a wavelength of 500 nm with good controllability. The fabrication process for the NCH material is scalable, cost-effective, and compatible with flexible substrates, offering promising opportunities for future applications.

#### 2.1.2. Advantages and Limitations of Inorganic-Based Materials

Inorganic-based flexible electrochromic materials offer a number of advantages and disadvantages that affect their suitability for various applications. Understanding these properties is critical to effectively exploit their potential. A notable advantage of inorganic-based materials is their stability [90]. They exhibit good chemical and electrochemical stability, which makes them highly reliable for long-term use in electrochromic devices. This stability ensures consistent performance over extended periods of time and improves durability under various environmental conditions. In addition, inorganic materials offer a wide range of colors [58]. This variety provides versatile design options for electrochromic applications and enables the fabrication of visually appealing and customizable displays, windows, and other optoelectronic devices. In addition, inorganic-based materials exhibit high optical contrast, which is essential for electrochromic applications [96]. They can significantly change their optical properties between colored and bleached states, allowing a clear distinction between on and off states, which improves their functionality and visual impact. Fast response times are another advantage of inorganic-based materials [84]. They can switch quickly, enabling rapid modulation of optical properties, which is critical for real-time adjustments and dynamic control in smart windows or display applications.

However, inorganic-based flexible electrochromic materials also have some disadvantages. One limitation is their limited flexibility compared to their organic counterparts [66]. Inorganic materials tend to be less flexible, which limits their suitability for applications that require high flexibility or conformability to curved surfaces. This limitation can lead to problems when integrating inorganic materials into certain devices or substrates. Another disadvantage is that some inorganic materials can have monotonous colors [42]. Certain inorganic materials may offer limited color choices, limiting the visual effects that can be achieved with electrochromic devices and compromising design options and the potential for customization in certain applications. In addition, the higher manufacturing costs of inorganic materials can pose a challenge [97]. Inorganic materials often require complex manufacturing processes or special equipment, resulting in higher production costs compared to organic materials. This cost factor can limit the scalability and application of flexible inorganic-based electrochromic technologies.

Ongoing research and development efforts are focused on overcoming these limitations. Strategies include improving the flexibility of inorganic materials by exploring novel material compositions, thin-film deposition techniques, or hybrid approaches to improve adaptability to flexible substrates and expand potential applications. Efforts are also aimed at expanding the color options of inorganic materials by exploring new materials, dopants, or alloying strategies to achieve a more vibrant and diverse color palette. To overcome slow switching speeds, researchers are investigating nanoscale engineering, surface modifications, or incorporation of additional materials to improve charge transport and enable faster electrochromic reactions. Advances in fabrication techniques, such as solution-based methods or roll-to-roll processes, can help reduce production costs and increase the scalability of flexible inorganic-based electrochromic devices.

### 2.2. Organic-Based Flexible Electrochromic Materials

**Figure 5:**
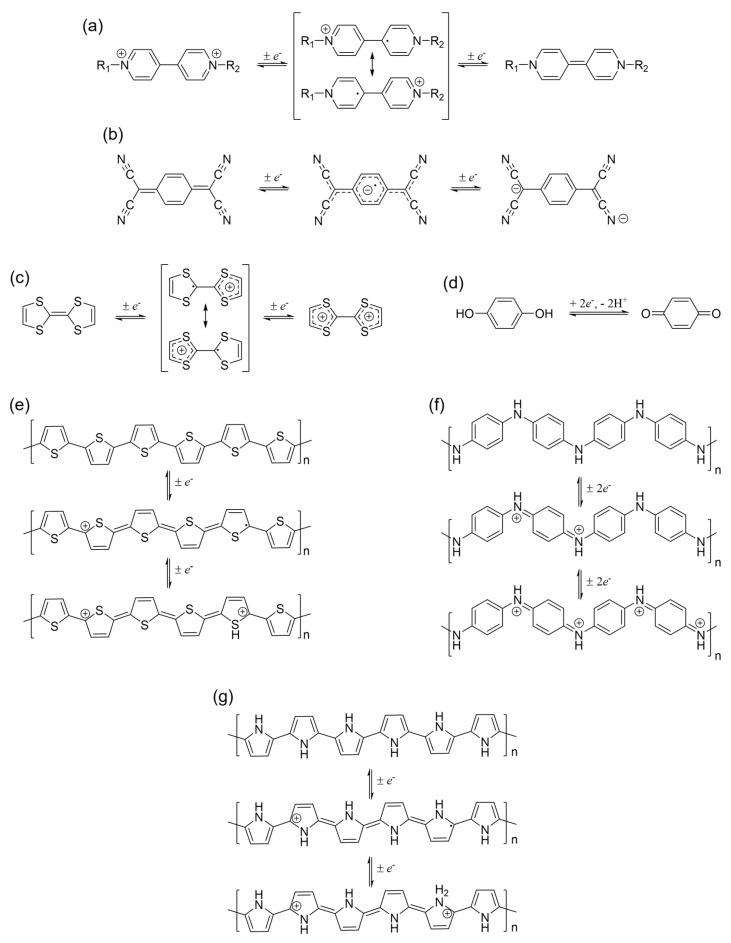
Redox states of various organic-based EC materials: (**a**) viologen derivatives (R_1_ and R_2_ are alkyl or aromatic moieties); (**b**) tetracyanoquinodimethane; (**c**) tetrathiofulvalene; (**d**) quinone; (**e**) polythiophene; (**f**) polyaniline; and (**g**) polypyrrole.

Recently, organic electrochromic materials have attracted considerable attention due to their numerous advantages over inorganic materials. These materials offer a wide range of bright colors, faster switching speeds, cost efficiency, and ease of processing [98]. Among the organic electrochromic materials studied, conductive polymers [99] and viologens [78] are the most commonly studied. Figure 5 shows various organic compounds, including viologen derivatives such as 1,1′-dialkyl-4,4′-bipyridiniumdications, tetrathiafulvalene (TTF) derivatives, tetracyanoquinodimethane (TCNQ) derivatives, quinones, and conductive polymers such as polythiophene (PTh), polyaniline (PANI), polypyrrole (PPy), and others [78]. A main feature of these materials is their chemical and electrochemical stability at applied electrical potential, leading to color changes between their oxidized and reduced states at relatively low potentials [100]. In addition, the structural makeup of these electrochromic materials significantly affects their light absorption at different wavelengths, making them suitable for low-power chromatic applications. In particular, viologen compounds have been extensively studied for their electrochromic properties, as they exhibit visual color changes at low operating voltages, provide high contrast and switchable electrochromism, and can be easily incorporated into cells [101].

Conductive polymers have attracted much attention in the field of flexible electrochromic materials due to their special properties and wide range of potential applications [102]. A major advantage of conductive polymers is their remarkable electrical conductivity. Unlike conventional organic materials, conductive polymers facilitate the flow of charges within the material, resulting in efficient electrochromic switching and modulation of optical properties [103]. This capability plays a critical role in the rapid and precise control of color changes in electrochromic devices. In addition, conductive polymers have the ability to undergo reversible redox reactions [104]. When an electric potential is applied, these polymers can change their electronic structure and show significant color changes. This property allows them to regulate light absorption and transmission, making them well suited for applications such as smart windows, displays, and optoelectronic devices. Another important advantage of conductive polymers is their flexibility [57]. They can be processed into flexible thin films or coatings, allowing seamless integration into flexible devices and substrates. This inherent flexibility not only facilitates the development of lightweight and adaptable electrochromic systems, but also improves the mechanical durability of the devices. Conductive polymers can be bent and stretched without compromising their electrochromic performance, making them ideal for wearable electronics and flexible displays.

#### 2.2.1. Examples and Case Studies of Organic-Based Flexible Electrochromic Materials

In a recent study by Moon et al. [105], they discovered that a random copolymer named poly[styrene-ran-1-(4-vinylbenzyl)-3-methylimidazolium hexafluorophosphate] (P[S-r-VBMI][PF_6_]) in combination with the ionic liquid 1-ethyl-3-methylimidazolium bis(trifluoromethylsulfonyl)imide ([EMI][TFSI]) can produce highly ionically conductive gels with remarkable mechanical robustness. Researchers used these gels to fabricate flexible electrochromic devices, benefiting from their excellent resistance to compressive and tensile stresses. Due to its versatility, the P[S-r-VBMI][PF_6_]-based gel has great potential for various flexible electrochemical applications, such as batteries and electrochemical displays.

A novel flexible electrochromic material suitable for wearable electronic devices was developed. For this purpose, a highly transparent, elastic, and conductive polyacrylamide hydrogel was used together with newly introduced water-soluble EC molecules such as thymol blue (Hydrogel-G) and phenol red (Hydrogel-R) sodium salts in combination with p-benzoquinone. This material offers the advantage of easily customizable and diverse color options and has excellent staining efficiency (291 cm^2^ C^−1^ and 152 cm^2^ C^−1^ for Hydrogel-R and Hydrogel-G, respectively) with minimal energy consumption. It also has health-promoting and environmentally friendly properties. Under tensile stress, the device can quickly and reversibly change its color from yellow to red or brown within seconds [106].

**Figure 6:**
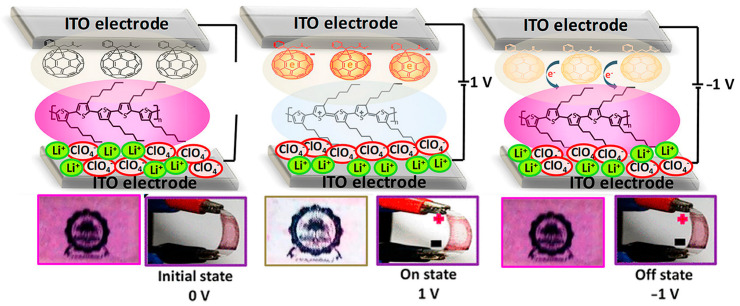
Illustration presenting the proposed mechanism governing the color transition in the electrochromic device. The diagram also features the flexible device itself, showcasing its response to various bias conditions. Adapted with permission from reference [107]. Copyright (2019), American Chemical Society.

Kumar and colleagues [107] describe the fabrication and performance of a flexible electrochromic device composed of purely organic materials. The device consists of layers of [6,6]-phenyl-C_61_-butyric acid methyl ester (PCBM) and poly(3-hexylthiophene-2,5-diyl) (P3HT) on a flexible substrate and features fast electrochromic switching with a switching time of 500 ms and a high staining efficiency of over 320 cm^2^/C. The device switches from a magenta state to a transparent state when a bias voltage of 1 V is applied. This indicates a redox switch of P3HT from its neutral state to a polaron state. If you reverse the polarity, the device returns to its magenta state. The PCBM molecules in the device act as electron storage and assist in the switching process. The device also shows good stability for 250 ON/OFF cycles and exhibits a high color contrast of over 50% and 90% efficiency in absorption switching. In addition, the ON state of the device shows increased IR absorption, indicating its potential application as a thermal filter in smart windows. Overall, the device represents an improvement in the field of fully organic electrochromic devices based on P3HT. The proposed switching mechanism is shown in Figure 6.

Viologen-based electrochromic materials have attracted considerable attention because of their exceptional properties, which make them highly desirable for various applications. These materials have special properties that distinguish them from others and make them an attractive choice in the field of electrochromics. One of their main advantages is the low operating voltage they require [108]. Viologen-based materials can undergo reversible redox reactions and exhibit significant color changes even at relatively low electrical voltages. This not only enables energy-efficient operation, but also reduces power consumption in electrochromic devices. Because they require less electrical energy, viologen materials contribute to the overall efficiency and sustainability of the devices in which they are used. In addition, viologen-based materials exhibit high contrast between their oxidized and reduced states. The color change observed in viologen films is visually obvious and allows for a clear and distinct change in optical properties [12]. This high contrast is crucial to achieve vivid and impressive visual effects in electrochromic applications. Whether it is smart windows, displays, or optical devices, the ability to produce significant changes in color and optical properties is a valuable attribute. Another remarkable property of viologen materials is their switchable electrochromic behavior [109,110]. They can switch between colored and bleached states in response to external stimuli. This property allows dynamic control of light absorption and transmission, which enables their application in various technologies. Although flexible electrochromic materials based on viologens offer numerous advantages, they also face certain challenges that require further research and development. Improving the long-term stability of viologen materials, especially under prolonged exposure to environmental factors, is an important focus [78,111]. Researchers are trying to improve their light and moisture resistance to ensure their reliability over extended periods of time. Efforts are also being made to improve the color range, efficiency, and longevity of viologen-based electrochromic devices to expand their applications and open new opportunities.

#### 2.2.2. Advantages and Limitations of Organic-Based Materials

Flexible organic-based electrochromic materials offer numerous advantages that make them highly desirable for a wide range of applications [14]. One of their main advantages is flexibility, as organic materials can naturally bend and conform to different surfaces without sacrificing functionality [107]. This inherent property allows the integration of these materials into flexible and bendable devices, making them ideal for applications where durability and flexibility are required.

In terms of energy efficiency, organic-based electrochromic materials require low voltage and low power consumption to achieve color changes [112]. This low power consumption is particularly advantageous for portable and wearable devices, where minimizing power consumption is critical to extending battery life.

In addition, organic materials can be chemically modified and tailored to achieve specific electrochromic properties [113]. By adjusting the molecular structure, composition, and doping process, the color change behavior, reaction time, and stability of organic materials can be precisely adjusted to meet the specific requirements of various applications. However, flexible organic-based electrochromic materials also have their limitations. One major drawback is their limited stability over time [114]. Organic materials are susceptible to degradation when exposed to environmental factors such as moisture [115], UV radiation [68], and temperature fluctuations [116]. The challenge of maintaining the long-term stability and durability of organic-based electrochromic devices remains an ongoing endeavor. Nevertheless, ongoing research and development efforts are focused on overcoming these limitations and improving the performance, stability, and functionality of flexible organic-based electrochromic materials. Advances in material design, surface engineering, protective coatings, and encapsulation techniques are being explored to improve the durability, response time, and color stability of these materials. With ongoing advances, organic-based electrochromic materials have the potential to be used in various applications that require flexible and efficient control of optical properties.

### 2.3. Hybrid and Composite Flexible Electrochromic Materials

Hybrid and composite electrochromic materials have emerged as promising options for flexible devices, offering enhanced electrochromic properties and improved overall performance. By effectively combining different components, these materials harness the strengths of each material, resulting in a wide range of benefits. One approach combines conductive polymers with nanoparticles [117], such as polyaniline or polythiophene. These nanoparticles can be metal oxides such as tungsten oxide or carbon-based materials such as graphene. The resulting hybrid materials exhibit improved electrochromic properties due to the enhanced charge transport and optical contrast provided by the nanoparticles, while maintaining the flexibility and processability of the conducting polymers. Another strategy is the preparation of composite materials by incorporating nanoparticles into polymer electrolytes [118]. Metal oxides or metal nanoparticles are commonly used nanoparticles in these composites. The presence of nanoparticles facilitates ion transport, resulting in faster reaction times and improved staining efficiency in electrochromic devices.

**Figure 7:**
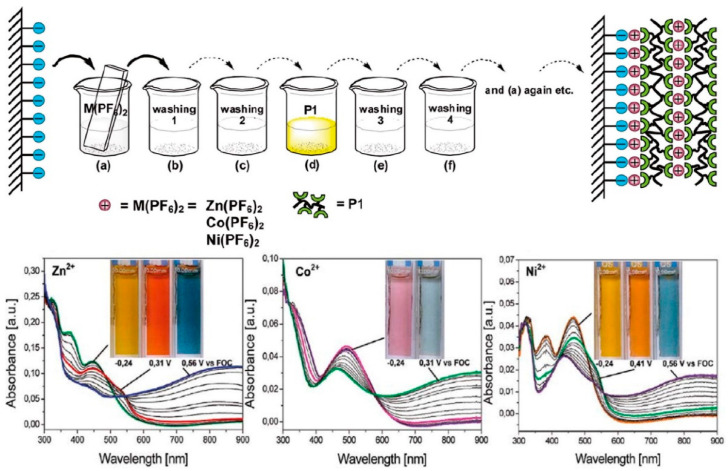
Coordination polymer films of metal(II) salts and P1 (poly(4-(2,2′:6,2″-terpyridyl)phenyliminofluorene)) were formed through sequential assembly. Spectroelectrochemistry was performed on these films using 12 dipping cycles on ITO-coated glass supports. Color transitions after 24 dipping cycles are shown in the inset pictures. The experiments were conducted using a 0.1 M TBAPF6/acetonitrile electrolyte/solvent couple at applied potentials (V). Adapted with permission from reference [119]. Copyright (2017), Elsevier.

Nanocomposites consisting of a polymer matrix in which nanoparticles or nanowires are embedded are another example of hybrid and composite electrochromic materials (Figure 7) [119]. The nanoparticles can be metal oxides or quantum dots. These materials provide greater surface area, enhanced charge transport, and better color stability, improving the overall electrochromic properties of the device. Hybrid films combining organic and inorganic components are also being explored [120]. Researchers can achieve tunable colors, improved stability, and flexibility in electrochromic devices by incorporating organic dyes or polymers into inorganic matrices such as metal oxides or metal–organic frameworks (MOFs). This combination of organic and inorganic materials provides a synergistic effect that improves electrochromic performance.

Furthermore, there has been significant attention directed towards graphene-based composites in the field of electrochromic materials [121]. Graphene, a two-dimensional carbon material, can be integrated into composites alongside other electrochromic materials. This integration offers several notable advantages, including enhanced electrical conductivity, mechanical strength, and optical properties. As a result, these composites are exceptionally well suited for flexible electrochromic devices. Moreover, organic–inorganic hybrid perovskites have emerged as a particularly promising class of materials for electrochromic applications [122]. These compounds possess exceptional optoelectronic properties. By incorporating hybrid perovskites into electrochromic devices, it becomes possible to achieve efficient color changes and high optical contrast. Overall, hybrid and composite electrochromic materials provide a compelling pathway for the development of flexible devices, offering improved performance, stability, and functionality. The combination of different materials leads to synergistic effects and enables the tailoring of properties to meet specific application requirements. With ongoing research and development efforts, hybrid and composite electrochromic materials hold tremendous promise for the future of flexible electrochromic devices.

#### 2.3.1. Examples and Case Studies of Hybrid and Composite Flexible Electrochromic Materials

Zhang et al. [123] used a novel approach in their study by combining a conductive polymer with silver nanowires. They fabricated composite films by layering electrochromic polymers (ECPs) and silver nanowires (AgNWs) on glass substrates coated with indium tin oxide, which served as electrodes in electrochromic devices. The incorporation of the AgNWs network into the composite films played a critical role in improving the overall performance of the ECDs. The presence of the AgNW network significantly improved several aspects, most notably the electrical conductivity of the films. This improvement facilitated the diffusion and migration of ions within the ECDs, promoted efficient ion doping, and optimized the electrochromic processes.

Numerous researchers have suggested that the integration of conjugated polymers with inorganic materials in nanocomposites leads to an improved electrochromic performance compared to using single conjugated polymers alone [124]. By introducing carbon nanotubes (CNTs) and graphene into the crystalline domains of conjugated polymers, the dyeing efficiency can be improved due to the bridging effect of the conductive nanoparticles, which reduces the charge transfer resistance. Furthermore, the incorporation of inorganic nanofillers such as metal oxides can create a highly porous structure, resulting in shorter diffusion lengths and enabling faster switching speeds. Several effective methods are used to fabricate conductive polymer nanocomposites in electrochromic applications.

Previous studies have shown that the combination of organic and inorganic materials in hybrid structures has advantageous properties, such as multicolor changes and high energy storage capacity, making them well suited for smart supercapacitor applications [125]. For example, an oblate WO_3_ morphology was synthesized by aggregating nanoclusters using hydrothermal methods. Subsequently, a hybrid material known as WO_3_-poly(5-cyanoindole) (P5ICN/WO_3_) was prepared via the electrochemical polymerization of P5ICN on the surface of WO_3_. The synergistic effect of P5ICN and WO_3_ in the hybrid material resulted in improved charge transfer rates, multiple color options (blue-green, yellow, and green), and favorable supercapacitive properties.

#### 2.3.2. Advantages and Limitations of Hybrid and Composite Materials

Hybrid and composite materials have attracted considerable attention in various fields due to their ability to synergistically combine different components and exploit their respective advantages. These materials offer a wide range of benefits that improve performance and functionality in various applications. A major advantage of hybrid and composite materials is their ability to improve properties [126]. These materials exhibit improved electrical conductivity, optical contrast, charge transport, and color stability by combining elements such as conducting polymers with nanoparticles or polymer electrolytes with nanoparticles. Consequently, they outperform their individual counterparts. For example, hybrid materials that combine conducting polymers with nanoparticles offer flexibility, processability, and enhanced charge transport and optical properties. In addition, these materials offer the advantage that their properties can be tailored. By tailoring the composition and structure, properties such as color range, reaction time, mechanical strength, and flexibility can be adapted to specific requirements. This capability enables the development of optimized materials for their intended applications [127]. Another notable advantage is the synergistic effect observed in hybrid and composite materials [125,128]. The interaction between different components leads to properties that exceed the sum of their individual parts, resulting in optimized performance and functionality in various applications. For example, the incorporation of nanoparticles or nanowires increases the surface area of the material, which enables enhanced charge transport and energy storage capacity in electrochemical devices. In addition, hybrid and composite materials are very versatile and find applications in various industries, such as electronics, energy storage, sensing, and optoelectronics [129]. The ability to combine different materials and explore diverse design possibilities further extends their utility.

However, it is important to be aware of the limitations associated with hybrid and composite materials [127]. The fabrication process can be complex and requires specialized equipment, controlled environments, and precise synthesis techniques. This complexity increases production costs and presents scalability challenges, especially for large-scale manufacturing. Stability and longevity are also important factors [130]. Compatibility and interactions between different components can affect the long-term stability of these materials, especially when they are exposed to environmental factors such as humidity, UV radiation, and temperature fluctuations. Despite these limitations, the challenges facing hybrid and composite materials are being actively addressed through ongoing research and technological advances. It is likely that the benefits of these materials will outweigh their limitations as researchers continue to innovate. This, in turn, will pave the way for broader application in various industries, contributing to technological advances and the development of more efficient and functional materials.

## 3. Fabrication Techniques for Flexible Electrochromic Materials

The advancement of flexible electrochromic devices requires the use of specialized fabrication techniques that allow seamless integration of electrochromic materials on flexible substrates while maintaining their mechanical integrity. Several approaches to fabricating flexible electrochromic devices have been investigated, each with different advantages and obstacles. In this section, we provide an overview of common methods for fabricating these devices, including solution-based techniques, thin-film deposition techniques, and print and roll-to-roll techniques.

### 3.1. Solution-Based Fabrication Techniques

**Figure 8:**
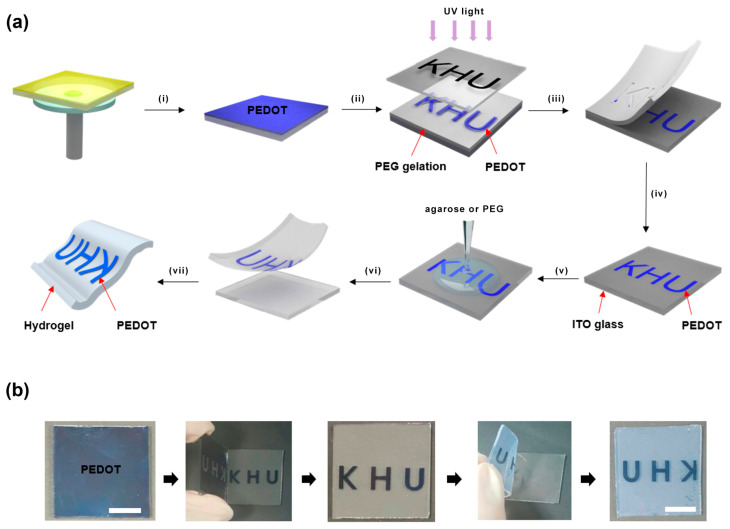
(**a**) Demonstration of conductive polymer pattern fabrication on various substrates using solution-based methods. Steps include (i) monomer casting and oxidative polymerization, (ii) photolithography, (iii, vi) peeling off layers, (iv) PEDOT in ITO glass, and (v) gelation processes. (vii) The resulting patterns are transferred from glass to a flexible hydrogel substrate. (**b**) Photographs depict the process, and scale is represented by a 1 cm bar. Adapted with permission from reference [99]. Copyright (2016), American Chemical Society.

Solution-based techniques include the deposition of electrochromic layers through methods such as spin coating, dip coating, or inkjet printing [97]. These techniques offer remarkable advantages in terms of their simplicity, cost-effectiveness, and scalability. The electrochromic materials are applied to the flexible substrate in the form of solvents or dispersions, followed by a curing or drying process that leads to the formation of the electrochromic layer. The choice of solvent and deposition parameters plays a critical role in determining the quality of the film and the performance of the device. Recent studies have proposed a novel sequential solution method for the preparation of poly(3,4-ethylenedioxythiophene) (PEDOT) patterns on various substrates, including hydrogels, as shown in Figure 8 [99].

The traditional methods involving multi-step chemical etching or lift-off processes are not required in this approach. In this experimental process, nano-thin PEDOT films were prepared on a glass substrate via the solution-phase casting of monomers and oxidative polymerization. A photomask was then used for UV-induced poly(ethylene glycol) (PEG) photolithography at the PEDOT/PEG interface. By detaching the hydrogel from the PEDOT-coated glass substrate, the UV-exposed PEDOT region was separated, leaving the unexposed PEDOT region intact on the glass substrate.

Researchers have successfully developed a scalable method to synthesize uniform films of electrochemically active amorphous metal oxide films such as WO_3_, Nb_2_O_5_, MoO_3_, and V_2_O_5_. This was achieved by a solution-based photodeposition technique [131]. Remarkably, these amorphous metal oxide films exhibit an exceptional electrochromic performance. The researchers propose this deposition technique as a fast, scalable, and cost-effective approach to effectively implement electrochromic windows.

### 3.2. Thin-Film Deposition Techniques

Thin films of electrochromic materials can be fabricated on flexible substrates using vacuum-based techniques such as physical vapor deposition (PVD) and chemical vapor deposition (CVD) [132,133]. In PVD processes such as sputtering or evaporation, the material is vaporized and then condensed on the substrate. CVD methods, on the other hand, use chemical processes to produce thin films. The vacuum-based processes provide precise control over film thickness, composition, and uniformity, improving device performance. However, the scalability and cost-effectiveness of these methods can be limited due to the need for high-vacuum conditions and the complexity of the equipment involved.

**Figure 9:**
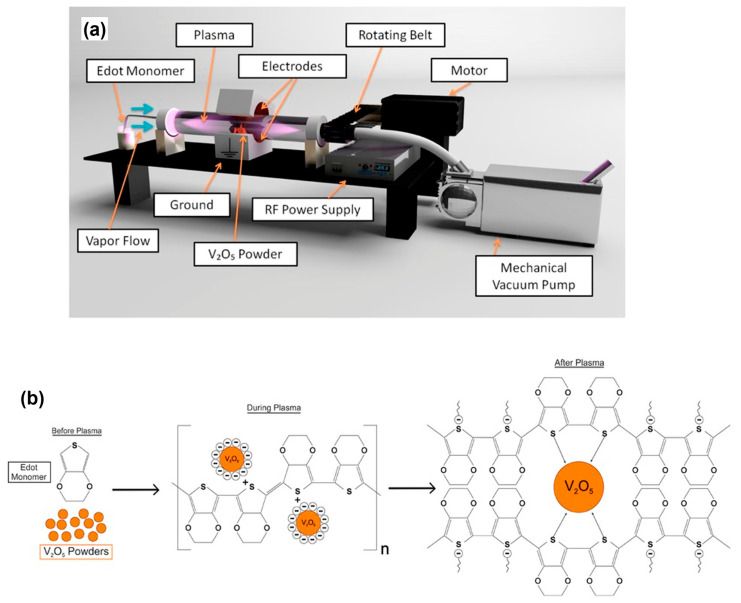
(**a**) A schematic diagram of RF rotating plasma, and (**b**) an illustration of the plasma polymerization process. Adapted with permission from reference [134]. Copyright (2017), John Wiley and Sons.

In one study, researchers used the electron beam evaporation technique to create thin films of a V_2_O_5_-PEDOT hybrid on a conductive PET/ITO film substrate with a sheet resistance of 80 to 100 Ω/sqm. By pelletizing V_2_O_5_ hybrid and pure V_2_O_5_ powder and subjecting them to electron beam heating, they successfully generated a uniform coating on the flexible PET substrate (Figure 9) [134].

Gomes et al. performed a study on the deposition of indium zinc oxide (IZO) on different types of paper using both radio frequency (RF) and direct current (DC) sputtering techniques [47]. A ceramic IZO target was used for the deposition process. By optimizing the deposition conditions, the researchers were able to produce high-quality thin IZO films on the papers. These films showed an exceptional performance with a low sheet resistance of about 20 Ω/sq and an impressive optical transmittance of about 80% in the visible spectrum range. Remarkably, these material properties remained stable for over eight months, indicating the long-term durability of the deposited IZO films.

### 3.3. Printing and Roll-to-Roll Techniques

Printing techniques such as screen printing and flexographic printing offer a flexible and cost-effective solution for the fabrication of flexible electrochromic devices [135]. In these techniques, electrochromic inks or pastes are applied to a substrate using a patterned stencil or flexible printing plates. Printing offers numerous advantages, including scalability, high resolution, and compatibility with various substrates. However, to achieve the desired electrochromic properties, it is critical to optimize ink formulations, ensure ink stability, and perform appropriate post-treatments.

**Figure 10:**
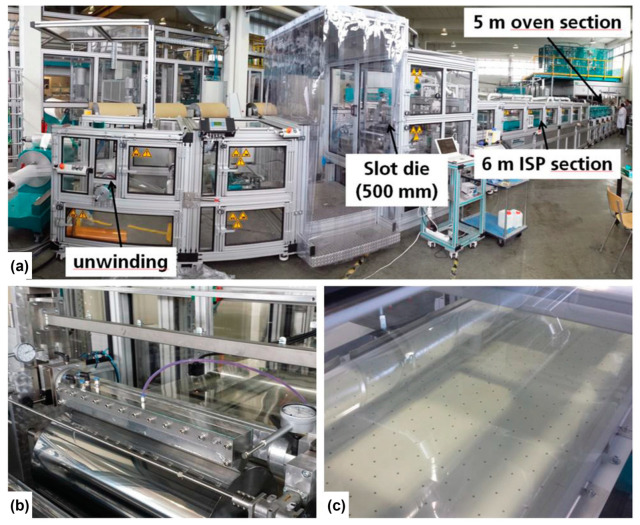
A modular roll-to-roll (R2R) coating machine enables the efficient deposition of PEDOT-EthC6 thin films onto PET-ITO substrates for large-scale production. (**a**) Modular R2R coating machine with unwinding unit, slot die, ISP, and oven section. (**b**) Slot die deposition. (**c**) ISP section (6 m), showing a yellowish colored wet film. Adapted with permission from reference [136]. Copyright: the authors, some rights reserved, exclusive licensee John Wiley and Sons. Distributed under a Creative Commons Attribution License 4.0 (CC BY).

To enable large-scale production of flexible electrochromic devices, roll-to-roll techniques have proven to be a promising approach (Figure 10) [136]. These techniques offer several advantages, such as high throughput, cost efficiency, and compatibility with flexible substrates. Most importantly, roll-to-roll processes are characterized by high production throughput. In contrast to conventional batch processes, roll-to-roll techniques enable continuous and fast production of electrochromic devices [137].

Roll-to-roll techniques offer significant advantages in the fabrication of electrochromic devices. In these techniques, a flexible substrate, such as a polymer film or metal foil, is continuously fed and subjected to a series of processing steps. This continuous processing enables efficient and simultaneous deposition, patterning, and encapsulation of electrochromic materials, resulting in a significant increase in production capacity. One of the main advantages of roll-to-roll techniques is their compatibility with flexible substrates, which allow the fabrication of pliable and stretchable electrochromic devices [69]. By maintaining a continuous flow, this process provides uniform deposition of electrochromic materials on flexible substrates without causing mechanical stress or deformation. This flexibility is critical for applications where electrochromic devices must conform to curved surfaces or be mechanically deformed. In addition, roll-to-roll technology can be used to deposit a variety of flexible substrates, such as polymer films, metal foils, and textiles, expanding the potential applications of flexible electrochromic materials [138].

## 4. Challenges and Future Perspectives of Flexible Electrochromic Materials

Although great progress has been made in the development of FECDs in recent years, there are still some difficulties to overcome in material preparation, structural optimization, device assembly, and practical application. For example, well-designed microstructures are desired to improve the EC performance of EC materials. A novel flexible conductive layer is required to improve device flexibility. The challenges in fabricating FECDs mainly include adhesion between active material layers, the chemical and mechanical stability of EC materials, and encapsulation of FECDs [34]. Here are some of the problems and their possible solutions:

- Adhesion problems are a major challenge for flexible electrochromic devices, as they often experience a significant decrease in staining efficiency with repeated bending. This decrease is mainly due to weak adhesion between the EC layer and the flexible substrate. While thermal treatment is commonly used to improve adhesion for rigid EC devices, it is unsuitable for flexible substrates due to their susceptibility to high temperatures. Therefore, it is crucial to develop stable EC films that can firmly adhere to flexible substrates without the need for thermal annealing [139]. This would eliminate the need for thermal treatment during the coating process and enable the fabrication of reliable and durable EC films for flexible devices. Finding alternative methods to achieve strong adhesion in flexible EC films is an urgent priority. For example, Lee and colleagues demonstrated the fabrication and analysis of transparent and stretchable AgNW-based transparent conductive electrodes for use in ECDs [37]. They achieved this by building a network of AgNWs on the surface using xenon flash techniques and employing silane surface treatments to promote strong adhesion. The study showed that the AgNWs effectively facilitated the conductive pathways. However, when applied without the silane surface treatment, bonding was impaired due to the contrasting hydrophilic nature of the NWs and the hydrophobic nature of the PDMS substrate.
- Material stability is one of the biggest problems, especially when using organic-based EC materials. The processes of dyeing and bleaching in electrochromic devices involve reversible introduction and the removal of ions and cause internal stresses during bending that lead to deformations in the microstructure of ECDs [91]. These deformations directly affect the electrochemical cycling stability of the materials. To address this problem, researchers have investigated various EC materials with different optical morphologies and structures, such as porous structures, two-dimensional nanosheets, and three-dimensional nanocolumns [38]. These structures facilitate charge transfer at the interface, ion penetration, and help to alleviate the internal stresses of the film during bending cycles. However, the electrochemical cycling stability of metal-based transparent conductive electrodes in flexible electrochromic devices, especially when silver electrodes are used, is poor due to oxidation and corrosion at positive potentials in the electrolyte. This oxidation and corrosion further contribute to the degradation of EC performance. To overcome the challenges during the transfer process and enable large-scale production of graphene-based transparent conductive films (G-TCFs), researchers have explored various modified transfer processes in the context of CVD methods [140]. For example, one approach is to investigate a carrierless transfer method to transfer CVD-grown graphene films over large areas onto various substrates modified with fluorine-containing self-assembling monolayers (F-SAM). This method involves floating the graphene films on the surface of a solution via a slow and nearly static process or using physical approaches to improve the adhesion between the graphene and the target substrates. These modifications aim to improve the efficiency and reliability of the transfer process to eventually enable industrial-scale production of G-TCFs.
- Encapsulation and protection are critical factors in maintaining the optimum performance of flexible electrochromic devices by protecting the active layers from environmental factors that can cause degradation, such as moisture and oxygen. To ensure the longevity and reliability of these devices, it is critical to develop robust encapsulation techniques specifically designed for flexible substrates. The use of encapsulation techniques plays a critical role in the practical fabrication of FECDs. In the device configuration, the electrolyte layer fills the space between the top and bottom electrodes, while the substrate, except for its edges, provides effective barrier properties for the entire device. However, the exposed portion of the active materials at the edges is more susceptible to permeation by oxygen and moisture, leading to oxidation and corrosion. Therefore, proper encapsulation is required to protect the active materials from these adverse effects. For example, Kim et al. [141] used a hydrothermal method to synthesize two-dimensional MoSe_2_ as a protective layer for AgNWs. This innovative approach led to the development of a flexible transparent conductive electrode (MoSe_2_/AgNWs/ PET) for flexible electrochromic devices. The structure PET/AgNWs/MoSe_2_/WO_3_/ EL-72/MoSe_2_/AgNWs/PET exhibited favorable properties, including a high dyeing efficiency of 37.47 cm^2^/C and an optical contrast of 42.14%.

Flexible electrochromic devices have great potential as materials of the future because they can overcome current challenges and have the potential for improvement and enhancement. Advances in material design, particularly in multichromic properties, and the development of multifunctional devices that integrate energy harvesting and storage bring FECDs to the forefront of technological innovation. To ensure long-term viability and sustainability, researchers must prioritize environmentally friendly and sustainable electrochromic materials by exploring green synthesis methods, using environmentally friendly components and optimizing device design to minimize a material’s environmental impact throughout its life cycle. However, more research is needed to overcome the challenges associated with large-scale applications, such as uniformity, scalability, and consistent performance over a large area. Overcoming these obstacles can facilitate the widespread adoption and integration of flexible electrochromic materials in industries such as smart windows, displays, energy-efficient buildings, and wearable electronics. In summary, the continuous improvement and expansion of FECDs combined with an environmentally friendly and sustainable approach will shape the future of this technology. It will revolutionize various sectors and contribute to an energy-efficient and environmentally conscious world.

## Data Availability

Data sharing not applicable.
